# Schizophrenia risk loci from xMHC region were associated with antipsychotic response in chronic schizophrenic patients with persistent positive symptom

**DOI:** 10.1038/s41398-022-01854-9

**Published:** 2022-03-07

**Authors:** Jiang Li, Akane Yoshikawa, Ney Alliey-Rodriguez, Herbert Y. Meltzer

**Affiliations:** 1 Department of Psychiatry and Behavioral Sciences, Northwestern University Feinberg School of Medicine, Chicago, IL USA; 2 Department of Molecular and Functional Genomics, Weis Center for Research, Geisinger Health System, Danville, PA USA; 3 Department of Psychiatry and Behavioral Sciences, Juntendo University Graduate School of Medicine, Tokyo, Japan

**Keywords:** Pharmacogenomics, Prognostic markers, Prognostic markers

## Abstract

We examined whether common variants from the extended major histocompatibility complex (xMHC) region contribute to the response to antipsychotic drugs (APDs) in patients with schizophrenia with persistent psychosis. Subjects participated in a prospective longitudinal study of the effect of APDs on psychopathology were temporally split into discovery (*n* = 88) and replication (*n* = 42) cohorts. The primary endpoint was a change in Brief Psychiatric Rating Scale at 6-week or 6-month after treatment. rs204991 (*β* = 3.917, *p* = 3.72 × 10^−6^), the strongest signal associated with response at 6-week was located near *C4A/C4B* after a linear regression adjusted for covariates. xMHC SNP imputation disclosed much stronger signals (rs9268469, *β* = 5.140, *p* = 1.57 × 10^−7^) and other weaker signals (*p* < 1 × 10^−5^) spanning the entire xMHC region. All the variants were previously identified schizophrenia risk loci. Conditional fine-mapping revealed three subgroups of SNPs which were the eQTLs (*p* < 1 × 10^−7^) for *C4A, HLA-C*, and *BTN3A2* in disease-relevant tissue. Epistasis between *HLA-C* and *C4A* was observed (*p* = 0.019). Minor allele (G) carriers of rs204991, eQTL for *C4A*, having decreased risk for schizophrenia and lower imputed expression of C4A, had a better response to APDs. Some imputed HLA alleles associated with a decreased risk for schizophrenia had a positive association with improvement in psychotic symptoms. An independent cohort validated the association of change in psychosis with *C4A*. We provide evidence that genetic risk factors for schizophrenia from the xMHC region are associated with response to APDs and those variants significantly alter the imputed expression of *C4A, HLA-C*, and *BTN3A2*. The minor alleles predicting higher C4A level are associated with diminished improvement in psychotic symptoms after APD treatment.

## Introduction

Schizophrenia (SCZ) is a complex syndrome affecting 1% of the population worldwide. There are likely diverse abnormalities during and after development underlying the positive, negative, and cognitive symptoms that characterize the illness. However, immune dysfunction and chronic inflammation have been of major interest in this regard [1]. Post-mortem morphological evidence, including loss of white matter and cortical gray matter without observed cell death [2], excessive synaptic pruning of mature or new dendritic spines in cortical pyramidal neurons [3, 4], and activation of microglia during adolescence and early adulthood [5], has led to the suggestion that these abnormalities may be due, in part, to immune system dysfunction. This may be the result of major histocompatibility complex (MHC) molecules (HLA I & II) mediated antigen-presentation and the complement-mediated classical pathway, producing microglial engulfment and auto-phagocytosis [6–10]. The hyperactivated microglia could be the major basis for excessive synaptic elimination [5, 11].

Functional implications of MHC genes in neuropsychiatric disorders, particularly schizophrenia, were strongly supported by genome-wide association studies (GWAS) which indicated that the extended MHC region (xMHC, a total of 7.6 Mb on the short arm of chromosome 6 with genetic coordinates between 25 M and 34 M) were the most significant and replicable genetic associations with SCZ [12–14]. A functional study showed that the level of C4A, a pivotal complement molecule, partially account for this association [7]. This landmark study integrated the genetic, autoimmune, and neurobiology (e.g., excessive synaptic pruning) theories of schizophrenia. It was proposed that overexpression of C4 in mouse prefrontal cortical neurons negatively affected dendritic spine development by the enhancement of microglia-induced synaptic engulfment and led to enhanced negative symptoms [15, 16]. Transcriptional imputation of SCZ GWAS summary statistics confirmed the increased expression of C4A, BTN3A2 from xMHC in several brain regions associated with schizophrenia, including dorsolateral prefrontal cortex (DLPFC) [7, 17].

There is some evidence that neuroinflammation has a significant impact on the course of schizophrenia. Significantly higher plasma levels of C3 and C4, as well as other acute-phase proteins have been reported in APD-treated compared to non-medicated SCZ patients [18]. A significant increase in cerebrospinal fluid C4 levels was observed in SCZ patients [19]. The HLA system has been linked to clinical response to the haloperidol [20] and chlorpromazine [21]. Although some single nucleotide polymorphisms (SNPs) at xMHC region predicted the treatment response to the atypical APDs, olanzapine, and risperidone [22], none of those markers have been linked to risk for SCZ or have a functional impact on alteration of gene expression at the xMHC region. C4A or C4B expression, imputed by copy number analysis of long and short forms of *C4A* and *C4B*, does not contribute to the risk and severity of tardive dyskinesia in SCZ patients [23]. There is a paucity of studies which have examined the role of C4 in treatment response to APDs in schizophrenia patients or other populations [24].

The purpose of this study was to identify common variants contributing to the treatment response to APDs in chronic schizophrenic patients in a candidate gene study of the xMHC region. The primary endpoint was the change in the subscales of the Brief Psychiatric Rating Scale (BPRS) at 6-week or 6-month after treatment with atypical APDs, including clozapine, olanzapine, risperidone, and lurasidone. A secondary fine-mapping approach was conducted to prioritize the potential causal genes associated with improvement in psychotic symptoms.

## Methods

### Subjects and clinical evaluation of treatment response

A structured interview, the Schedule for Affective Disorders and Schizophrenia [25], or the Structured Clinical Interview for Schizophrenia for DSM III or IV [26], provided the basis for diagnosis. This was integrated with all available data to make the final diagnosis by consensus according to the Diagnostic and Statistical Manual of Mental Disorders, third edition (DSM-III) criteria at discharge. A review of these diagnoses indicates all patients met DSM-IV criteria for schizophrenia or schizoaffective disorder. Data from patients with either diagnosis were combined. Over 75% of patients were unmedicated or had a drug-free period of 3–10 days prior to baseline assessment.

All participants were temporally split into discovery (*n* = 88) and replication (*n* = 42) cohorts. Demographics and clinical information for the discovery and replication cohorts were provided in Table 1. For quantitative variables, Shapiro–Wilk test confirmed the approximately normally distributed data for these variables (*p* > 0.05) and Welch’s *t*-test was conducted to compare the mean difference between two cohorts with an assumption of equal variance or unequal variance determined by Levene’s test; for categorical variables, Fisher’s exact test was conducted to examine the significance of the association between two kinds of classification. The subjects selected for GWAS had participated in a prospective clinical trial of the effect of atypical APDs. Diagnosis and classification as treatment-resistant schizophrenia (TRS) or non-treatment-resistant schizophrenia (NTRS) were based on severity of positive symptoms and poor functional outcome after two or more trials of APDs of usually adequate duration [27]. Patients whose psychotic symptom responded to atypical APDs other than clozapine were classified as NTRS. Determination of TRS or NTRS was based upon review of medical records for inpatient and out-patient treatment and assessment of response of positive symptoms to two or more trials with ADPs, including the current trial [28].

**Table 1:** Demographic, psychopathology, and genotype data for discovery and replication cohorts.

Cohort	Discovery (*n* = 88)	Replication (*n* = 42)	Statistics (*p* value)
Gender (M/F)	64/24 (72.7%/27.3%)	31/11 (73.8%/26.2%)	1
Age of onset	21.07 ± 7.19	20.07 ± 7.43	0.464
Diagnosis (SCZ/SAD)	75/13	38/4	0.58
TRS/NTRS	55/33 (62.5%/37.5%)	35/7 (83.3%/16.7%)	0.025
Antipsychotic drugs	CLZ/RISP/OLZ	CLZ/RISP/OLZ/ZIPR/LURA	<2.2e−16
	60/13/15 (68.2%/14.8%/17.0%)	1/19/6/2/14 (2.4%/45.2%/14.3%/4.8%/33.3%)	
BPRS_BASE	14.5 ± 9.95	12.7 ± 10.66	0.348
BPSY_BASE^a^	10.98 ± 3.09	7.5 ± 3.69	1.07E−07
WR_BASE	4.53 ± 2.67	4.21 ± 3.17	0.549
∆BPSY_6wk	2.98 ± 3.89	1.14 ± 2.75	0.002
∆BPSY_6mon	3.90 ± 3.70	3.62 ± 3.44	0.689
∆WR_6wk	0.65 ± 2.70	0.57 ± 2.30	0.869
∆WR_6mon	1.00 ± 2.92	1.22 ± 2.23	0.674
Array (Illumina)	610 K quad BeadChip	PsychArray	NA
# of SNPs after QC	491,932	272,589	
# of SNPs at xMHC (genotyped + imputed)	42,763 (3722 + 39041)	40,684 (2707 + 37977)	

Data were presented as mean ± SD. Most of the patients were initially hospitalized for an acute exacerbation of chronic psychosis. Only patients with baseline BPSY ≥ 6 were included in this analysis. These patients should be described as having persistent moderate to severe psychotic symptoms which were technically all TRS. Regional SNP imputation was conducted at xMHC by IMPUTE2 using 1000 Genome Project samples (2014) as reference.

*SCZ* Schizophrenia, *SAD* Schizoaffective disorder, C*LZ* clozapine, *RISP* risperidone, *OLZ* olanzapine, *ZIPR* ziprasidone, *LURA* lurasidone, *xMHC* extended major histocompatibility complex, *TRS* treatment-resistant schizophrenia.

^a^BPRS positive symptom subscale, refers to as BPSY, includes suspiciousness, hallucinatory behavior, and unusual thought. BPRS negative subscale, WR, is comprised of three items: emotional withdrawal, motor retardation, and blunted affect. Quantitative treatment response was evaluated at 6-week and 6-month, using the change in ∆BPSY or ∆WR. Welch’s *t*-test or Fisher’s exact test was conducted to compare the difference between two cohorts for the quantitative or categorical variables, respectively, and the corresponding *p* value was present.

The discovery GWAS included 88 self-described Caucasian patients diagnosed with schizophrenia or schizoaffective disorder by DSM-IV criteria. They were recruited between 1999 and 2010 at clinical facilities associated with Departments of Psychiatry at Case Western Reserve University and Vanderbilt University. Most of these patients were initially hospitalized for an acute exacerbation of symptoms and failure to respond adequately to conventional or atypical antipsychotic drugs. The subjects selected for GWAS had participated in a clinical trial (NCT00539071, NCT00179062) or prospective longitudinal studies of the effect of clozapine (*n* = 60), olanzapine (*n* = 15), or risperidone (13). 62.5% patients in the discovery cohort were classified as TRS [29]. An additional 42 subjects with were studied at Vanderbilt University and Northwestern University [30]. The subjects selected for GWAS had participated in a clinical trial (NCT01569659, NCT00179062) or prospective longitudinal study of the effect of lurasidone (*n* = 14), risperidone (*n* = 19), olanzapine (*n* = 6), clozapine (*n* = 1), or ziprasidone (*n* = 2).

Here we use the classical 18-item Brief Psychiatric Rating Scale (BPRS) with 0 to 6 scaling for each item. All ratings were conducted by trained neuropsychiatric technicians who were blind to the hypothesis of this study. The BPRS positive symptom subscale, BPSY, includes assessment of suspiciousness, hallucinatory behavior, and unusual thought content. The BPRS negative subscale, WR, which stands for BPRS negative subscale, is comprised of three items: emotional withdrawal, motor retardation, and blunted affect. Quantitative treatment response was evaluated at 6-week and 6-month, using the change in ∆BPSY or ∆WR. Only patients with moderate to severe psychosis (baseline BPSY ≥ 6) were included in the analysis. Different cutoff values for BPSY had been implemented to avoid selection bias, and BPSY ≥ 6 gave the strongest signal compared to BPSY ≥ 0, ≥2, ≥4, and ≥8 (see the Supplementary Table 1 for effect size and power analysis), with 88 out of previously reported 174 patients [29] included in the discovery cohort. Therefore, the same baseline cutoff values were applied to the replication cohort. 42 out of 71 patients were included.

After a description of the study, written informed consent was obtained from every subject. All patients provided written informed consent to remain drug-free during the assessment. The drug-free period was terminated if patient well-being required it. Some were not receiving psychotropic drugs prior to admission because of non-compliance. This study was approved by institutional review boards from Case Western Reserve University, Vanderbilt University, and Northwestern University.

### Quality control of genotyping data and association testing

Genome-wide SNP genotyping was performed using Illumina 610 K quad BeadChip^®^ for discovery cohort [29] or Illumina PsychArray^®^ for replication cohort [30].

DATA QC was conducted to exclude samples with minor allele frequency (MAF) < 0.05, genotyping rate per SNP < 0.95, and significant deviation from Hardy–Weinberg equilibrium (*p* < 0.0001). There were 491,932 (Discovery) and 272,589 (Replication) SNPs available for further analysis. Total genotyping rate in the remaining individuals was >99.96%.

Regional SNP imputation for xMHC (Chr6: 25 M to 34 M) was conducted by IMPUTE2 using 1000 Genome Project (April 2014) as reference panel. Briefly, after prephasing by SHAPEIT2 using 37 macGT1 data as reference, we conducted a stepwise imputation in 5-Mb segment using Quest High Performance Computing Cluster. The imputed data was finally converted to PLINK format by GTOOL. SNPs with imputation quality core >0.9 were used in the following association testing. All cases were considered as unrelated individuals (PiHAT = 0.20 as a cutoff) based on the pairwise identity-by-descent (IBD). All patients (discovery and replication) recruited in this study were self-described Caucasians which was verified by Principal Component Analysis (PCA) [29, 30].

The association testing was conducted by PLINK 1.9. The primary endpoint was change in psychotic (ΔBPSY) or negative (ΔWR) symptoms of BPRS at 6-week and 6-month after treatment with APDs. Linear regression in an additive model of minor alleles, adjusted for covariates, first three principal components (PC_1-3_), gender, and drug, was utilized. TRS status was tested as a covariate in the linear regression model but had no significant impact on the association for the top variants. Therefore, it was not included as a covariate in the summary statistics. False discovery rate (FDR) corrections for multiple testing were calculated using the Benjamin and Hochberg (BH) procedure for the regional association testing.

Mapping cis eQTL in disease-related tissues was conducted by three online portals, LIBD eQTL browser (http://eqtl.brainseq.org/) [31], GTEx Portal (https://gtexportal.org/home/), and scanDB (http://scandb.org/newinterface/about.html). We arbitrarily considered *p* < 4 × 10^−5^ as the cutoff for cis-eQTL, providing >25,000 genes which could be investigated.

Transcriptome imputation and gene-based association testing was conducted by PrediXcan [32] using the PredictDB, GTEx-V7_HapMap-2017-11-29.tar.gz, solely from subjects of European ancestry (EUR) to impute gene expression at xMHC (Chr6: 25 M to 34 M). The dosage file for our samples (Discovery and Replication) was created by PLINK. Linear regression adjusted for covariates such as first three principal components(PC_1-3_), gender, and drug, was utilized to determine a gene-level association from imputed expression to ΔBPSY or ΔWR. A raw *p*-value was calculated. The Benjamini–Hochberg procedure was conducted to adjust the false discovery rate (BH-FDR) from each brain tissue and the *q* value <0.1 was considered significant.

Classical MHC I & II and amino-acid polymorphisms were imputed by SNP2HLA (version 1.02) [33] using T1DGC_REF from The Broad Institute as reference and association testing was performed to determine if the candidate HLA alleles, previously reported to increase risk for SCZ, also contributed to the variation in treatment response/resistance.

Finally, Targets Genetics (www.OpenTargets.org), a PheWAS portal, was employed to prioritize the causal variants with functional impact and explore their pleiotropy if any.

See Supplementary material for additional description of the methods. The summary statistics of our GWAS may be shared with the third party upon execution of data sharing agreement for reasonable requests. The analysis codes are available upon request.

## Results

Table 1 summarized the demographic, psychopathology (baseline and change after 6wk or 6mon), as well as microarray information for discovery (*n* = 88) and replication (*n* = 42) cohorts, respectively. It was noted that baseline, as well as the improvement in psychotic symptoms at 6-week in the replication cohort, were significantly lower than those in the discovery cohort; the type of APDs was also quite different. These factors contributed to reducing the power of replication of the top findings identified in the discovery cohort.

The initial attempt to identify genetic variants associated with improvement in psychotic symptoms (∆BPSY) at 6-week after treatment with APDs was made by GWAS in a cohort of chronic schizophrenic patients of European ancestry (EUR) with persistent psychotic episodes (*n* = 88). Manhattan and QQ plots were shown in Supplementary Fig. 2. No genome-wide significant loci were identified after a linear regression adjusted for covariates, suggesting the complexity of the trait and that this GWAS was underpowered because of small sample size. Nevertheless, rs204991 (genotyped, but not imputed, *β* = 3.917, *p* = 3.72 × 10^−6^), was the 2nd genome-wide strongest signal. It, and other weaker signals, was noteworthy because of its location near C4A/C4B. TRS status was tested as a covariate in the linear regression model but has no significant impact on the association for the top variants (for rs204991, *p* = 0.64; for rs6904596, *p* = 0.91). The medication was not the significant covariate in the genotype-phenotype association for the top hit, rs204991 (*p* = 0.488 and *p* = 0.193 in both discovery and replication cohorts). However, we retained it as a covariate in the full regression model.

**Fig. 1:**
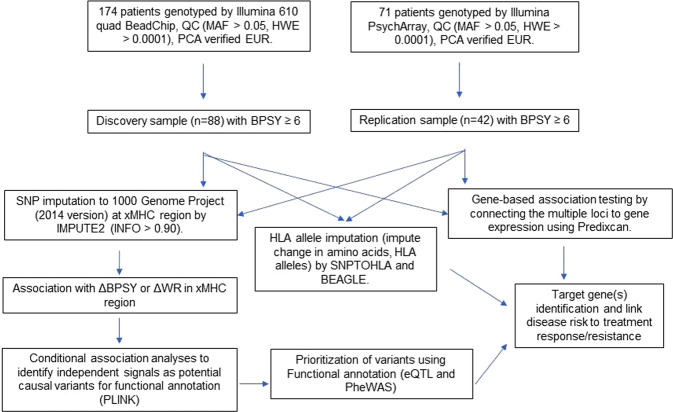
Overview of the strategy for the fine-mapping causal variants/genes from xMHC region in association with treatment response to APDs. ΔBPSY, change in positive symptom subscale including suspiciousness, hallucinatory behavior, and unusual thought content; eQTL, expression quantitative trait loci; EUR, European ancestry; HWE, Hardy–Weinberg equilibrium; INFO, an information score, which takes a value between 0 and 1, reported by IMPUTE2, with a value near 1 indicating high certainty of imputed genotype; MAF, minor allele frequency; PCA, principal component analysis; QC, quality control; PheWAS, phenom-wide association studies; ΔWR, change in withdrawal symptom subscale which is comprised of three items: emotional withdrawal, motor retardation and blunted affect.

The subsequent fine-mapping by a candidate region approach (Fig. 1) was conducted after regional SNP imputation in order to identify causal variants or genes from xMHC associated with treatment response to APDs. SNP imputation disclosed much stronger signals with *β* = 5.140, *p*_lowest_ = 1.57 × 10^−7^ for rs9268469 and other weaker signals (*p* < 1 × 10^−5^) spanning the entire xMHC region.

**Fig. 2:**
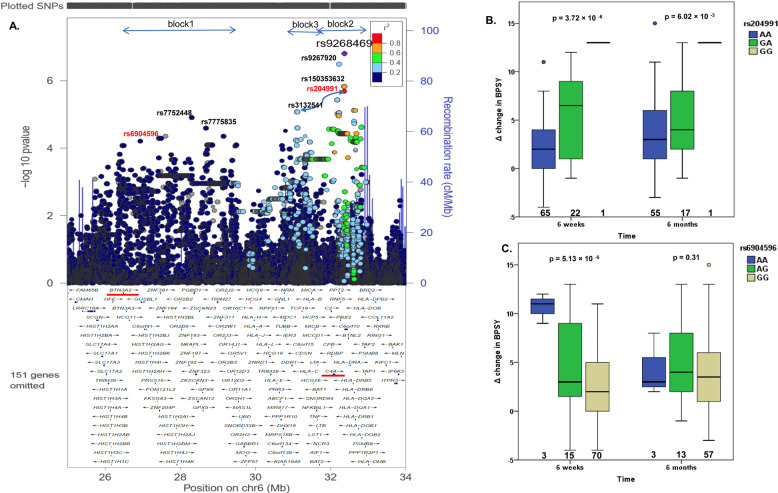
The genetic variants at xMHC were associated with improvement in psychotic symptoms at 6-week after treatment with APDs. **A** Common SNPs (maf > 0.05) within xMHC region were genotyped or imputed to 1000 Genome Project reference panel (April 2014) with high confidence (INFO > 0.90). The linear regression adjusted for the covariates including gender, drug, and PC 1–3 derived from the initial GWAS were conducted by PLINK to determine the association between SNPs and treatment response to APDs. The regional association plot was created by LocusZoom. Purple diamond indicated the most significant finding at this region. Chromosomal positions and LD were based on hg19/1000 Genome Project (April 2014) EUR. Colors represent LD (*r*^2^) with the top SNP, rs9268469. Based on the LD pattern, the top signals at xMHC can be partitioned into three blocks. Estimated recombination rate (light blue line) was plotted on the right y-axis. The top signals of real-typed SNPs from the representative LD blocks were labeled in red font. The epistasis testing of a set of SNPs with *p* < 10^−4^ in the linear regression model were conducted and the top SNP×SNP was the interaction between rs204991 and rs3132541 (BETA_INT = 5.24, *p* = 0.019). **B**, **C** The boxplots showed the distribution of ∆BPSY at 6-week or 6-month stratified by each genotype of rs204991 (**B**) and rs6904596 (**C**). Minor allele (G) carriers of rs204991 having decreased risk for SCZ and lower expression of C4A had a better response to clozapine in BPSY at both 6-week and 6-month after treatment with APDs; minor allele (A) carriers of rs6904596 having decreased risk for SCZ had a better response to clozapine in BPSY only at 6-week after treatment with APDs.

The top signals associated with improvement in psychotic symptoms at 6-week were labeled in the regional association plot (Fig. 2A) and listed in Table 2 after LD-based clumping. We also listed the results from two genotyped SNPs, rs6904596 and rs7775397 which were in LD with two imputed SNPs, rs150353632 and rs59134830. The association of top SNPs with symptom improvement at 6-month was tested. Only SNPs (rs204991, *β* = 2.57, *p* = 0.006, Fig. 2B) near *C4A*, but not those closest to *BTN3A2* (rs6904596, *β* = 0.9, *p* = 0.309, Fig. 2C) showed a significant association (*p* < 0.01) but with smaller effect size (*β*).

**Table 2:** Summary of the top genetic variants or genes at xMHC regions in association with symptom improvement.

SNP_ID	SNP information (LD clumped)				1KG	Discovery (610 K QUAD)							Replication (PsychArray)			PGC_GWAS			Discovery (610 K QUAD)	
						∆BPSY_6wk				∆BPSY_6mon			∆BPSY_6wk						∆WR_6wk	∆WR_6mon
	BP_hg19	NSIG-S05-S01-S001-S0001	Minor/major	MAF	MAF	*N*	BETA	*P*	FDR-_BH	*N*	BETA	*P*	*N*	BETA	*P*	A1/A2	OR	*P*	*P* (*n* = 42)	P (*n* = 36)
rs9467772	26496578	0-24-0-0-4(28)	T/A	0.176	0.19	88	2.907	8.40E−05	0.013	73	0.92	0.25	42	−0.16	0.83	A/T	1.13	1.85E−18	0.189	0.793
rs147925578 (rs2770573)	26937830	0-0-10-0-0(10)	C/A	0.178	0.137	87	3.256	6.22E−05	0.013	72	0.939	0.29	NA	NA	NA	A/C	1.13	1.43E−16	0.723	0.806
rs144022448	27456052	0-0-6-41-6(53)	C/CAG	0.119	0.081	88	3.358	5.13E−05	0.013	73	0.9	0.31	42	1.437	0.15	NA	NA	NA	0.956	0.344
***rs6904596***	27491299	LD with rs59134830	A/G	0.119	0.082	88	3.358	5.13E−05	0.013	73	0.9	0.31	42	1.437	0.15	A/G	0.88	4.60E−19	0.956	0.344
rs59134830 (rs35442355)	27607111	0-0-1-8-1(10)	TA/T	0.213	0.153	87	2.839	4.43E−05	0.013	72	1.323	0.08	41	1.091	0.19	NA	NA	NA	0.662	0.449
rs7752448	28301099	0-0-0-1-0(1)	G/A	0.114	0.093	88	4.089	1.24E−05	0.013	73	0.849	0.43	42	0.46	0.63	A/G	1.19	1.92E−21	0.846	0.628
***rs13194504 (rs116521087)***	28630691	LD with rs1233579	A/G	0.097	0.069	88	3.34	1.12E−03	0.04	73	0.076	0.95	41	1.988	0.07	A/G	0.95	3.51E−30	0.928	0.364
rs7775835	28678357	0-0-0-12-0(12)	T/C	0.136	0.106	88	3.521	2.56E−05	0.013	73	1.099	0.23	42	0.04	0.96	T/C	0.82	1.12E−28	0.665	0.568
***rs1233579***	28712663	0-2-82-7-95(186)	G/A	0.102	0.071	88	3.879	8.11E−05	0.013	73	0.853	0.43	42	1.391	0.16	A/G	1.25	1.06E−29	0.774	0.577
rs9257566	29144532	0-0-9-25-0(34)	T/C	0.131	0.091	88	3.456	4.45E−05	0.013	73	1.099	0.23	42	0.689	0.42	T/C	0.79	1.77E−30	0.665	0.568
rs2523432	29484110	0-0-17-1-4(22)	A/G	0.102	0.083	88	3.879	8.11E−05	0.013	73	0.853	0.43	42	0.676	0.47	A/G	0.81	4.78E−26	0.774	0.577
rs3094128	30694374	0-0-4-2-0(6)	C/T	0.142	0.163	88	2.952	8.40E−05	0.013	73	1.33	0.09	42	1.495	0.09	NA	NA	NA	0.529	0.374
rs886423	30782205	0-41-32-1-1(75)	C/G	0.095	0.131	84	4.392	3.07E−05	0.013	70	2.611	0.02	42	1.968	0.03	C/G	0.86	1.31E−16	0.661	0.651
rs3132541	31098734	0-3-41-111-11(166)	C/A	0.085	0.085	88	4.719	8.46E−06	0.013	73	2.078	0.07	42	1.406	0.19	A/C	1.17	3.71E−19	0.384	0.435
rs3094013	31434366	0-1-6-43-8(58)	A/G	0.075	0.084	87	4.557	6.61E−05	0.013	72	1.732	0.17	41	1.818	0.09	A/G	0.85	2.15E−19	0.623	0.632
***rs1150752***	32064726	0-1-1-19-10(31)	C/T	0.074	0.076	88	4.771	2.73E−05	0.013	73	1.402	0.27	42	1.774	0.08	T/C	1.17	9.53E−18	0.637	0.640
***rs204991 (rs114002920)***	32161366	0-3-2-0-4(9)	C/T	0.136	0.167	88	3.917	3.72E−06	0.013	73	2.57	0.01	42	1.118	0.24	T/C	1.12	1.10E−15	0.551	0.802
rs9267920	32206243	0-0-0-0-10(10)	T/C	0.108	0.13	88	4.873	3.25E−07	0.005	73	2.467	0.03	42	1.666	0.05	T/C	0.87	1.60E−17	0.434	0.561
***rs7775397 (rs114384056)***	32261252	LD with rs150353632	G/T	0.068	0.079	88	4.837	3.73E−05	0.013	73	1.828	0.16	42	2.091	0.03	T/G	1.18	4.07E−17	0.9313	0.217
rs150353632	32346463	0-3-5-1-58(67)	A/ATTTTGTG	0.08	0.086	88	5.238	1.49E−06	0.009	73	2.91	0.01	NA	NA	NA	NA	NA	NA	0.741	0.130
rs9268469	32353590	0-3-1-0-1(5)	A/G	0.103	0.107	87	5.14	1.57E−07	0.005	72	3.327	0	39	1.99	0.04	NA	NA	NA	0.922	0.716
rs9281989	32602030	0-1-3-7-46(57)	GAAGT/G	0.063	0.083	87	5.402	7.61E−06	0.013	72	2.41	0.07	40	1.956	0.06	NA	NA	NA	0.931	0.217
rs9272770	32610226	0-0-0-2-1(3)	A/G	0.103	0.15	87	4.311	1.74E−05	0.013	72	2.427	0.03	41	1.508	0.05	A/G	0.86	3.75E−13	0.589	0.081
rs60045856	32799845	1-0-12-2-1(16)	G/T	0.074	0.079	88	4.779	2.40E−05	0.013	73	2.369	0.05	42	0.473	0.54	T/G	1.15	5.77E−15	0.538	0.137

Summary statistics of top variants at xMHC region (25M–34M) with *p* value <1 × 10^−4^ in the discovery dataset were LD clumped (-clump-p1 1 × 10^−4^; -clump-*r*^2^ 0.5) and listed here. The number of SNPs clumped at each level of *p* value for those SNPs were also listed. Their association with ∆BPSY at 6-week in the replication dataset were also listed. Only SNPs originally genotyped but not imputed were in bold and italic font. Original and FDR-BH corrected *p* value was provided for real-typed or imputed SNPs. SNP ID in parentheses represented the alias name which were in line with the SNP ID in PGC GWAS in SCZ. BP was genomic coordinate based on hg19 version. We also listed the results from two real-typed SNPs, rs6904596 and rs7775397 which were in LD with rs150353632 and rs59134830, respectively. BETA always represented regression coefficient of the minor allele. OR always represented odds ratio for the effect size of A1 allele in PGC GWAS dataset. Only SNP and gene association with *p* < 0.0001 in subjects with European Ancestry were listed here. PGCGWAS data was collected from http://www.med.unc.edu/pgc/results-and-downloads.

### Conditional linear regression analysis to identify independent signals

Based on the LD pattern between rs9268469 and other weaker signals with *P*_FDR-BH_ < 0.05 (Fig. 2A), we partitioned the regional association plot into 3 blocks, block 1 (*r*^2^ < 0.2), block 3 (0.2 < *r*^2^ < 0.4), and block 2 (*r*^2^ > 0.4). In order to identify the independent effects, three representative SNPs which showed high LD (rs2240991, *r*^2^ > 0.8), intermediate LD (rs3132541, 0.2 < *r*^2^ < 0.4), and poor LD (rs6904596, *r*^2^ < 0.2), with rs9268469, from the corresponding three subregions, were selected as the fixed effect and added to the original linear regression model (Supplementary Fig. 3). The strength of the association of rs9268469 with treatment response in BPSY was attenuated from *β* = 5.14 (*p* = 1.57 × 10^−7^) to *β* = 3.99 (*p* = 0.001) after adjusting for rs3132541 (Supplementary Fig. 3A), suggesting the associations from rs3132541 and that from rs9268469 were not completely independent from each other. This was also found with rs6904596 (*β* decreased from 3.36 to 1.79; *p* changed from 5.13 × 10^−5^ to 0.06) and others in block one (Supplementary Fig. 3C) which spanned Histones and BTN3A2. The strength of the association of SNPs in block one or block two were only slightly changed after adjusting for rs6904596 (for rs9268469, *β* changed from 5.14 to 4.13, *p* changed from 1.57 × 10^−7^ to 1.09 × 10^−4^; for rs204991, *β* changed from 3.92 to 3.02, *p* changed from 3.72 × 10^−6^ to 4.44 × 10^−4^) respectively, suggesting that the top signals from block one were independent of those from block two. However, they both show significant interaction with signals from block three, suggesting SNP×SNP interactions.

Summary of the epistasis among the top 22 variants reported with the association *p* value <1 × 10^−5^ was provided in Supplementary Fig. 4A. We added β_3_g_snp1_g_snp2_ into the original linear regression model, *Y* = β_0_ + β_1_g_snp1_ + β_2_g_snp2_ + β_covar1_Covar1 + … for each inspected variant pair (SNP1, SNP2). The eQTL analysis from LIBD eQTL Browser (Supplementary Fig. 4B) indicated that rs3132541 and rs886423 have significant impact on the gene expression of HLA-C in DLPFC. Given the epistasis between rs3132541 and rs204991 (*p* = 0.019) or rs886423 and rs204991 (*p* = 0.033), the genetic evidence supported that there is a significant interaction between HLA-C and C4A. It was noted that all these variants show genome-wide significant risk for SCZ according to PGCGWAS on SCZ (Table 2A).

None of the top hits showed association with negative symptom improvement. An independent cohort (*n* = 42) validated the genetic association (rs9268409 and rs7775397) at C4A (*p* < 0.05) with improvement in psychotic symptoms in the same direction for the minor allele.

### eQTL analysis of the top variants in disease-related tissues

**Fig. 3:**
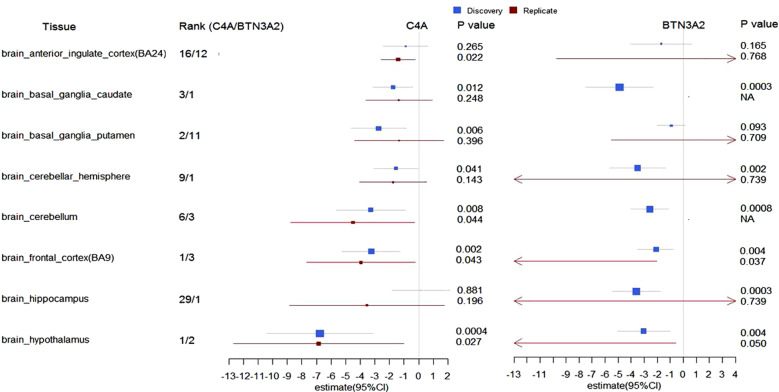
The forest plot illustrated the inverse association between the imputed expression level of C4A or BTN3A2 and treatment response (∆BPSY at 6-week) to atypical APDs in several brain tissues using PrediXcan. Transcriptome prediction model was GTEx-V7 (EUR only) from PredictDB based on elastic net; xMHC (Chr6: 25 M to 34 M) was the target region. Dosage file for our samples was created by PLINK. PrediXcan was run at Python 2.7. The estimates of effect size (*β*) with 95% confidence interval in discovery and replicate datasets were present, grouped by the brain region. The significance of this association was present as raw. The genes predicted to have the most significant impact on improvement in psychotic symptoms at 6-week after the treatment with APDs were ranked in each brain tissue. *C4A* (left) and *BTN3A2* (right) which represents one of the genes from block 1 and 2, has been frequently ranked to the top in schizophrenia-related tissues such as frontal cortex, hypothalamus, and basal ganglia (Caudate and Putamen). C4A level could not be successfully imputed using the transcriptomic data from “brain_basal_ganglia_nucleus_accumbens”. The result from Nucleus Accumbens was not included.

The boxplots from LIBD eQTL Browser on the left indicate the significant impact of top variants in block two on the gene expression of C4A in DLPFC (Supplementary Fig. 5A–C). The barplots on the right represent the significant impact of those variants on expression of C4A in multiple brain tissues and whole blood (Supplementary Fig. 5D, E). rs9268469 was a strong eQTL for C4A in those tissues (*p* = 5.5 × 10^−20^ in whole blood; *p* = 1.5 × 10^−8^ in hippocampus) according to GTEx. The boxplots in Fig. 3B showed the distribution of ∆BPSY at 6-week or 6-month stratified by each genotype of rs204991 (A) and rs6904596 (B). Minor allele (G) carriers of rs204991 have decreased risk for SCZ, lower expression of C4A, and a better response in BPSY at both 6-week and 6-month after treatment with APDs. Minor allele (A) carriers of rs6904596 have decreased risk for SCZ and a better response in BPSY only at 6-week after treatment with APDs.

### Transcriptome imputation and gene-based analysis

Prediction of gene expression in a variety of brain tissues based on the genetic architecture at xMHC was conducted to correlate imputed gene expression with ∆BPSY at 6-week using PrediXcan. The expression levels of nearly 29 to 37 genes from the xMHC were imputed; those numbers varied depending on brain tissue. The genes predicted to have the most significant impact on symptom improvement in BPSY were ranked in each brain tissue (Fig. 3). C4A and BTN3A2 from the corresponding block 1 and 2, have been frequently ranked at the top in some schizophrenia-relevant brain regions such as frontal cortex, basal ganglia, and hypothalamus. According to the forest plot (Fig. 3), patients predicted to have a higher level of C4A or BTN3A2 showed poor response to APDs, as indicated by the negative value of *β*. Significant associations were identified in frontal cortex and hypothalamus.

### HLA imputation and association testing

The most significant association between HLA alleles and ∆BPSY at 6-week is HLA_DRB1_0301 (*p* = 3.732 × 10^−5^). When including the top HLA allele reported in earlier PGC GWAS 2009 for the risk of SCZ, the presence of those HLA alleles which decreased risk for SCZ have significant positive association with improvement in psychotic symptoms at 6-week (Supplementary Table 2). HLA_DQA1_0501 is the only one not showing contribution to the treatment response. Some of those associations were also replicated (HLA_DRB1_0301, *p* = 0.023) or showed a trend of replication (HLA_DQB1_0201, *p* = 0.074) in the 2nd cohort. This is the first evidence for a connection between these SCZ risk-associated HLA alleles and treatment outcome in a subgroup of chronic schizophrenic patients with persistent moderate to severe positive symptoms.

## Conclusion

The purpose of this study was to identify common variants contributing to treatment response to APDs in chronic schizophrenic patients with persistent psychotic episodes using xMHC fine-mapping approach. Although no genome-wide significant locus was identified after linear regression adjusted for covariates (*n* = 88), rs204991 (*p* = 3.72 × 10^−6^), located near C4A/C4B, was among the top-tier signals. Regional SNP imputation disclosed even stronger signals with the lowest *p* = 1.57 × 10^−7^ for rs9268469 and other weaker signals (*p* < 1E−5) spanning the entire xMHC region. All these variants show genome-wide significant risk for SCZ. The subsequent LD-based conditional fine-mapping revealed three subgroups of SNPs which were found to be eQTLs (*p* < 1 × 10^−7^) for C4A, HLA-C, and BTN3A2 in disease-associated tissue such as DLPFC, basal ganglia, and whole blood. Genetic evidence of epistasis between HLA-C and C4A was observed (*p* = 0.019). Minor allele (G) carriers of rs204991, eQTL for C4A, who had decreased risk for SCZ and lower expression of C4A, had a better response to APDs at both 6-week and 6-month; Minor allele (A) carriers of rs6904596, eQTL for BTN3A2, who had decreased risk for SCZ have a better response to APDs only at 6-week. In line with these findings, HLA imputation showed the presence of some HLA alleles which decrease the risk for schizophrenia [12] have a positive association with improvement in psychotic symptoms. An independent cohort (*n* = 42) validated the genetic association between response to APDs and the C4A expression. These top SNPs associated with treatment response in schizophrenia were also related to several autoimmune-related diseases (e.g., coeliac disease, hyperthyroidism/thyrotoxicosis). Neuroinflammation could be a part of systemic dysfunction of immune system in SCZ. A recent meta-analysis confirmed an overall positive association between non-neurological autoimmune disorders and psychosis with a larger effect size (odds ratio) in pernicious anemia, pemphigoid, psoriasis, celiac disease, and Graves’s disease [34]. Prolonged inflammatory/immune response may contribute to treatment resistance in some schizophrenic patients [24, 35, 36]. Together, we provide evidence that some genetic risk factors for SCZ in the xMHC region are associated with treatment response/resistance to APDs and those variants significantly alter the gene expression of C4A, HLA molecules (HLA-C), and other immune-related genes (BTN3A2).

### Linking the genetic risk for disease to treatment outcome or other intermediate phenotypes

The initial attempts to map SCZ risk loci to the MHC region were first reported in 1974 [20]. Since then, many reports provide additional evidence [37] but the definitive evidence for MHC involvement in SCZ was the result of three GWAS of SCZ patients with EUR descent published in 2009 [12–14]. One of the most significant association signals, rs13194053 with *p* = 9.54 × 10^−9^, was located at the extended MHC region. Others including rs6932590 (*p* = 1.4 × 10^−12^), rs1635 (*p* = 7 × 10^−12^), rs2523722 (*p* = 2.88 × 10^−16^), rs2021722 (*p* = 4.3 × 10^−11^), rs886424 (*p* = 4.54 × 10^−8^), rs3131296 (*p* = 2.3 × 10^−10^), and rs9272219 (*p* = 6.88 × 10^−8^) [9, 38, 39]. It was noted that all these SNPs showed association with improvement in psychotic symptoms in this study, e.g., rs13194053 (*β* = 2.065, *p* = 0.012); rs6932590 (*β* = 1.225, *p* = 0.056); rs1635 (identified from Han Chinese); rs2523722 (*β* = 0.761, *p* = 0.047); rs2021722 (*β* = 1.536, *p* = 0.047); rs3131296 (*β* = 4.726, *p* = 3.11 × 10^−5^) and rs9272219 (*β* = 1.256, *p* = 0.060). rs3131296 was closest to C4A and had significant impact on the gene expression of C4A (DLPFC *p* = 2.01 × 10^−18^). These genetic risk variants were associated with brain structure and cognition [38, 40]. Six SNPs show nominally significant association with one or multiple domains of cognitive function. The (G) allele carriers of rs6904071, which were associated with increased risk for SCZ, poor delayed episodic memory, and decreased hippocampal volume, showed poor response to APDs in our study (Supplementary Table 3). Other SNPs also showed the association with treatment response in the same direction.

### Chronic neuroinflammation may determine the patient outcome after treatment with APDs

According to the PheWAS report from UK Biobank (Supplementary Table 4), the top SNPs associated with treatment response in this study were related to several autoimmune-related diseases (e.g., coeliac disease, hyperthyroidism/thyrotoxicosis). Those SNPs were linked to many autoimmune diseases unrelated to neuropsychiatric phenotypes (Supplementary Table 5). The neuroinflammation could be a part of systemic dysfunction of immune system. Thus, we suggest as have others [34, 41] screening for some autoimmune disorders (autoantibodies) in patients who do not response to APDs.

Multiple lines of evidence support the relationship between neuroinflammation and schizophrenia. Polygenic risk scores for SCZ were higher in patients with early-life complications (ELCs) [42]. Genes whose expression was modified by ELCs were involved in regulation of oxidative and cellular stress as well as inflammation. A meta-analysis of RNAseq and array-based transcriptome study of lymphoblastoid cell lines derived from schizophrenia cases and controls have indicated immune-related genes as the top-ranked for differential expression [43].

Our study was based on a candidate region, followed by LD-based conditional association testing, which was extended to eQTL analysis, transcriptomic imputation and association, HLA imputation and association, and PheWAS. These efforts confirmed the association between the gene expression and response to APD treatment, but also prioritized the causal SNPs/Genes. It is, thus, quite noteworthy that C4A related SNPs had the most significant and replicable association with APD response. This suggests that chronic neuroinflammation not only contributes to the risk for development of psychosis, but also is important for predicting some types of improvement with atypical APD treatment. This finding also provides additional impetus for further study of anti-neuroinflammatory drugs as adjuvants to atypical APDs [44, 45].

The contribution of the MHC to negative symptom improvement was not supported by this study, although there was some evidence that chronic neuroinflammation may contribute to negative symptoms [46].

It is noteworthy that the C4 and HLA-related SNPs which predicted response in this study did not predict treatment response in acutely psychotic, NTRS patients who were included in double-blind registration trials of lurasidone [47, 48]. This suggests that the genetic predisposition for immune activation in schizophrenia may be most important for SCZ patients with chronic psychosis [49]. Only some brain regions in SCZ may be vulnerable to inflammation. Expression of inflammatory genes may be confined to specific brain region, as a transcriptomic study found that abnormal immune/inflammatory responses were limited to the hippocampus [50]. The hippocampal regional gene expression showed above suggests neuroinflammation may be important for the neurocognitive deficits associated with schizophrenia. Another study focusing on inflammatory genes in psychotic patients under or over 40 years of age found a difference in gene expression only in older patients [51]. The decreased expression levels of altered inflammatory genes in DLPFC in post-mortem specimens from aged SCZ correlated with the microglial marker CD68 [52]. This supports the idea of a dysfunction of these processes in aged patients and a possible relationship with active microglia abundance.

### C4A, synaptic plasticity, and effect of antipsychotic drugs

C4A copy number variation is associated with synaptic pruning. C4-deficient mice have shown decreased synaptic pruning [7]. Increased imputed *C4A* mRNA levels predicted poorer performance on memory recall and reduced cortical activity in middle temporal cortex during a measure of visual processing, suggesting that there is a positive correlation between predicted C4A transcription and impairment in memory [53]. A recent study on the changes in neuronal membrane expansion and contraction within the neuropil by phosphorus magnetic resonance spectroscopy has shown that C4A copy number positively correlated with neuropil contraction in the DLPFC and thalamus of adolescent-onset SCZ patients [54].

In addition, other immune-related molecules such as certain HLA I and II alleles, members of immunoglobulin superfamily, participate in the C4-mediated classical pathway, which is actively involved in activity-dependent synaptic remodeling and plasticity [55]. Identification of genetic variants which altered the expression of those molecules in this study suggests the activated autoimmune-mediated processes contribute to resistance to APDs.

### Limitations

The individual genetic associations reported here need to be validated by independent studies with larger sample sizes, similar open trial design and patient cohort, conducted by other investigators. Given the main effect of β_G_ (~3.83 for top markers like rs204991), a type 1 error rate of 1 × 10^−4^ for nominal significance with two-sided test, on the continuous trait with mean ± SD of ∆BPSY as 2.98 ± 3.89, we conducted a power test using QUANTO. Our sample size of 88 had >80% power to identify a significant association when MAF was equal to 0.136. Our study had limited power to test the association in the small replication cohort (*n* = 42). The levels of C4 and other proinflammatory cytokines in plasma or cerebrospinal fluid was not tested during or after the trial. We did not examine comorbidity information related to autoimmune or infectious diseases. Finally, our study was based exclusively on subjects of European ancestry. We did include a diversity of chronic schizophrenic patients with regard to treatment resistance status and current exacerbation of positive symptoms.

The major finding of this manuscript indicated that the genetic liability (i.e., xMHC region) to schizophrenia also contribute to the risk for treatment response to antipsychotic agents in a cohort of chronic schizophrenic patients with persistent positive symptom. This study highlights the importance of personalized treatment of schizophrenic patients, particularly for the subgroup of patients who might developed the treatment-resistant phenotype due to the persistent neuroinflammation, and further emphasizes the target treatment of this neuroinflammation as well as potential inflammation-related comorbidities. Although a recent prediction study by leveraging ‘real-world’ EHR data reported a poor prognostic value of SCZ polygenic risk score (PRS) for adults with psychosis, we hypothesize that this PRS may have a potential value in outcome prediction in a subgroup of patients of the similar kind as we reported in this study.

In conclusion, we demonstrate for the first time that genetic variants which predict higher C4A levels, an indicator of inflammation, are associated with less improvement in psychotic symptoms during treatment with APDs.

## Supplementary information

supplementary methods

supplementary figures and tables
