# Supplementary material for: Schizophrenia risk loci from xMHC region were associated with antipsychotic response in chronic schizophrenic patients with persistent positive symptom
Source: Transl Psychiatry. 2022 Mar 7;12:92. doi: 10.1038/s41398-022-01854-9 (PMC8898944; doi:10.1038/s41398-022-01854-9)
Supplement: Supplementary file 1 — supplementary methods [file 41398_2022_1854_MOESM1_ESM.pdf]

Schizophrenia risk loci from xMHC region were associated with antipsychotic response in chronic schizophrenic patients with persistent positive symptom

Supplementary material for method.

2.1 Mapping cis eQTL in disease-related tissue was conducted by three databases

LIBD eQTL browser (http://eqtl.brainseq.org/)

eQTL inquiry via LIBD eQTL browser is based on the RNA-seq and genotyping data collected from the BrainSeq phase 1 tissue cohort which consists of three main psychiatric diagnostic groups (schizophrenia, major depression, and bipolar disorder) of 738 postmortem human brain samples from the dorsolateral prefrontal cortex (DLPFC)[1]. The data publicly available were generated from mixed ancestry, mainly EUR.

GTEx Portal (https://gtexportal.org/home/)

The Genotype-Tissue Expression (GTEx) project is an ongoing effort to build a comprehensive public resource to study tissue-specific gene expression and regulation. Samples were collected from 53 non-diseased tissue sites across nearly 1000 individuals, primarily for molecular assays including WGS, WES, and RNA-Seq. Remaining samples were available from the GTEx Biobank. The GTEx Portal provides open access to data including gene expression, QTLs, and histology images. The current release is V7 including 11,688 samples, 53 tissues, 714 donors, mainly EUR.

scanDB (http://scandb.org/newinterface/about.html)

Transcriptomic analysis of 176 HapMap lymphoblastoid cell lines derived from individuals of EUR were conducted using the Affymetrix GeneChip (GSE9701). The genetic contribution to transcript isoform variation was then evaluated by a genome-wide association using the HapMap genotypic data on single nucleotide polymorphisms (SNPs). Only SNP and gene association with $p < 0.0001$ in subjects with EUR were listed in **Table 2**.

2.2 Transcriptome imputation and gene-based association testing

In many cases, multiple loci in poor LD with each other may have independent cis effects on the nearby gene expression. Modeling this genetic architecture in prediction of expression in neighborhood genes, knowns as transcriptome imputation, has been recently developed. Basically, using machine learning approach such as elastic net implemented by PrediXcan[2] to construct the model by connecting the local genotyping data with the level of gene expression

nearby. Then applying this established predictive model to imputation of the gene expression level of unknown samples but with known genotyping data. Finally, testing the association between the imputed gene expression data with the phenotype data of interested in order to identify the causal gene responsible for biological mechanisms underlying these associations. GTEx provides expression data obtained from multiple tissues including the tissue relevant to the phenotype of interest. This prediction model (PredictDB) can be built based on tissue of interest and ethnicity dependent. In this study, we only use the PredictDB, GTEx-V7_HapMap-2017-11-29.tar.gz, solely from subjects of EUR to impute gene expression at xMHC (Chr6: 25M to 34M). Dosage file for our samples (Discovery and Replication) were created by PLINK.

2.3 HLA imputation and association testing

Statistical imputation of classical HLA alleles based on SNPs is challenging and indispensable for fine-mapping of phenotype associated signals from GWAS. SNP2HLA[3] is an imputation software package built under BEAGLE for the purpose of impute HLA alleles and amino acid polymorphisms. HLA imputation by this method in eight HLA classes (HLA-A, HLA-B, HLA-C, HLA-DRB1, HLA-DQA1, HLA-DQB1, HLA-DPA1, and HLA-DPB1) showed high-level of accuracy (80%-98%). The amino acid changes within HLA genes might cause variations in the binding affinity of encoding HLA proteins. However, the exact underlying mechanisms behind those changes in contribution to the disease susceptibilities or drug efficacy/side effect remains unknown. Genotypes of xMHC region from our samples (Discovery or Replication) were first phased by BEAGLE (version 3.0.4) and Classical MHC I & II and amino-acid polymorphisms were imputed by SNP2HLA (version 1.02) using T1DGC_REF (kindly provided by The Broad Institute) as reference. This reference panel was constructed based on genotyping data collected from individuals of EUR. When testing for association with imputed classical HLA alleles, SNP2HLA defined a series of binary markers coding the presence or absence of the allele being tested, and each different allele was tested as a biallelic position just like a SNP. The output dosage files for the HLA alleles and change in amino acid were used for the following association testing. The linear regression assuming an additive model for minor allele, adjusted for the covariates including gender, drug, and PC1-3 derived from the initial GWAS were conducted by PLINK to determine the association between HLA alleles or AA polymorphisms and treatment response to APDs. The original p value was provided without correction for the multiple testing as the purpose of this study is to determine if the candidate HLA alleles, previously reported to have the increased risk for SCZ, also contributed to the variation of treatment response/resistance.

2.4 PheWAS to prioritize the causal variants and explore their biological implications

PheWAS is an alternative approach to prioritize causal genes at a trait-associated locus given allocation of nearest genes to the locus is a poor basis of gene annotation. Open Targets Genetics (www.OpenTargets.org) is a portal highlighting variant-centric statistical evidence to allow both prioritization of candidate causal variants at trait-associated loci, and identification of potential drug targets. This portal integrated cross-trait colocalization analyses and merges genetic associations curated from both literature and newly acquired association results from UK Biobank, which contains functional genomics data (e.g. chromatin conformation, chromatin interactions) and quantitative trait loci (e.g. eQTLs from GTEX, pQTL). PheWAS will be used for validation of the causal SNPs/Genes by expanding the connection between the disease risk and outcome prediction.

Reference:

1. Jaffe AE, Straub RE, Shin JH, Tao R, Gao Y, Collado-Torres L *et al.* Developmental and genetic regulation of the human cortex transcriptome illuminate schizophrenia pathogenesis. *Nat Neurosci* 2018; **21**(8)**:** 1117-1125.

2. Gamazon ER, Wheeler HE, Shah KP, Mozaffari SV, Aquino-Michaels K, Carroll RJ *et al.* A gene-based association method for mapping traits using reference transcriptome data. *Nat Genet* 2015; **47**(9)**:** 1091-1098.

3. Jia X, Han B, Onengut-Gumuscu S, Chen WM, Concannon PJ, Rich SS *et al.* Imputing amino acid polymorphisms in human leukocyte antigens. *PLoS One* 2013; **8**(6)**:** e64683.
