# Supplementary material for: Schizophrenia risk loci from xMHC region were associated with antipsychotic response in chronic schizophrenic patients with persistent positive symptom
Source: Transl Psychiatry. 2022 Mar 7;12:92. doi: 10.1038/s41398-022-01854-9 (PMC8898944; doi:10.1038/s41398-022-01854-9)
Supplement: Supplementary file 2 — supplementary figures and tables [file 41398_2022_1854_MOESM2_ESM.pdf]

Supplementary Figure 1. Principal Component Analysis of GWAS sample for Discovery and Replication. Regional SNP imputation was conducted by IMPUTE2 using 1000 Genome Project phase 1 (ALL_1000G_phase1integrated_feb2012_impute) as reference for the dataset collected by Illumina 610K quad BeadChip® or Illumina PsychArray®. Briefly, after prephasing by SHAPEIT2 using 37 macGT1 data as reference, we conducted a step-wise imputation in 5-Mb segment. The imputed data derived from two array platforms was converted to PLINK format by GTOOL and finally merged. Principal Component Analysis (PCA) was performed on autosomal SNP data of GWAS samples by PLINK (Supplemental Figure 1). The samples from Discovery (n=174) and Replication (n=71) cohorts against 1,092 individuals from 1000 Genome Phase 1 data (AFR, n=246; AMR,n=181; ASN, n=286; EUR, n=379). ~133521 SNPs from autosomes were originally genotyped and shared by Illumina 6q, PsychArray, and 1000 Genome sequencing data.

Supplementary Figure 2. Manhattan(2A) and QQ (2B) plots for GWAS results associated with ∆BPSY at 6-week after treatment with APDs in schizophrenic patients with European ancestry. Only patients with moderate to severe psychosis (Baseline BPSY ≥ 6) were included in the following analysis. The linear regression adjusted for the covariates including gender, drug, and PCs(1-3) from PCA were conducted by PLINK. Top variants with original p value < 10^{-5} were labeled. The arrow pointed to the candidate region at chromosome 6 for the fine-mapping. All SNPs included in these plots were genotyped, not imputed. The genomic inflation factor, λ_{GC}, equals to 1.0037, suggesting no evidence for systematic inflation of genome-wide test statistics.

Supplementary Figure 3. The regional association plot illustrated the potential dependent or independent signals from the result of conditional linear regression analyses. In order to identify the independent effects, three representative SNPs which showed high (rs2240991, $r^2 > 0.8$), intermediate (rs3132541, $0.2 < r^2 < 0.4$) , poor (rs6904596, $r^2 < 0.2$), in LD with the lead variant, rs9268469, from three subregions respectively, were selected as the fixed effect added to the original linear regression model.

Supplementary Figure 4. The summary of the epistasis among the top 22 variants reported with the association p value < 1×10^{-5}. We added $\beta_3 g_{snp1} g_{snp2}$ into the original linear regression model, $Y = \beta_0 + \beta_1 g_{snp1} + \beta_2 g_{snp2} + \beta_{covar1}Covar1 + \ldots$ for each inspected variant pair (SNP1, SNP2). The covariates included gender, drug, and PCs (1-3) derived from the GWAS. The β_3 coefficient, BETA_INT, were tested for significance. Only variant pairs with p < 0.05 for β_3 coefficient were listed. The eQTL analysis from LIBD eQTL Browser indicated that rs3132541 and rs886423 have significant impact on the gene expression of HLA-C. Given the epistasis between rs3132541 and rs204991 or rs886423 and rs204991, the genetic evidence support that there is a significant interaction between HLA-C and C4A.

Supplementary Figure 5. eQTL analysis of the top variants in disease related tissues. The boxplots (5A/5B/5C) on the left represent the significant impact of top variants in block two on the gene expression of C4A in DLPFC. The data was collected from LIBD eQTL Browser. The barplots (5D/5E) on the right represent the significant impact of those variants on gene expressions, particularly C4A in multiple brain tissues and whole blood. The data was collected from GTEx portal. Only p < 1×10^{-6} were listed here. No significant eQTLs were found for SNP rs9268469 in those tissues. All evidence supported that minor allele (C) carriers of rs204991 having lower expression of C4A had a better response to APDs in BPSY.

Supplementary Figure 1.

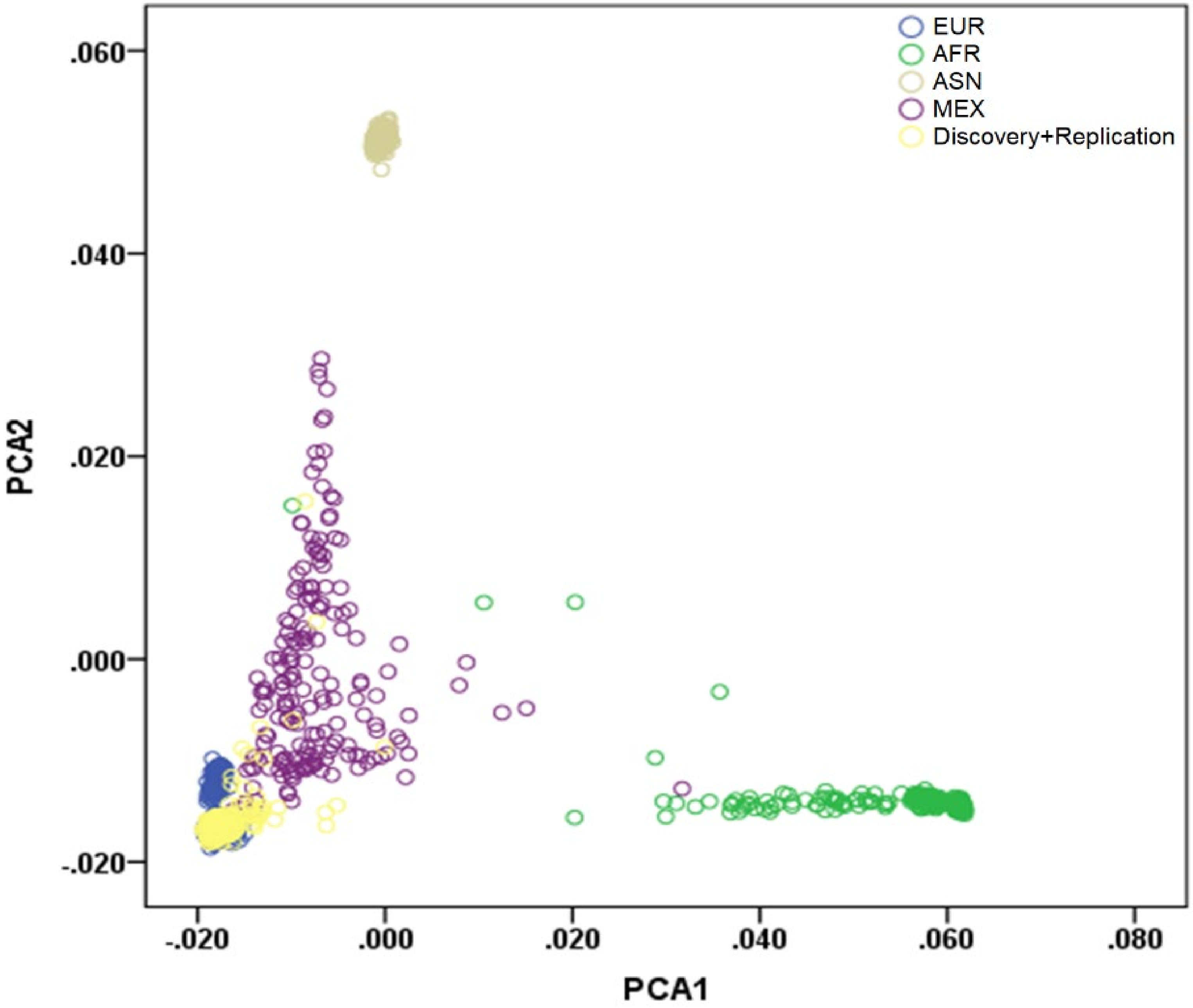

Supplementary Figure 2.

2A.

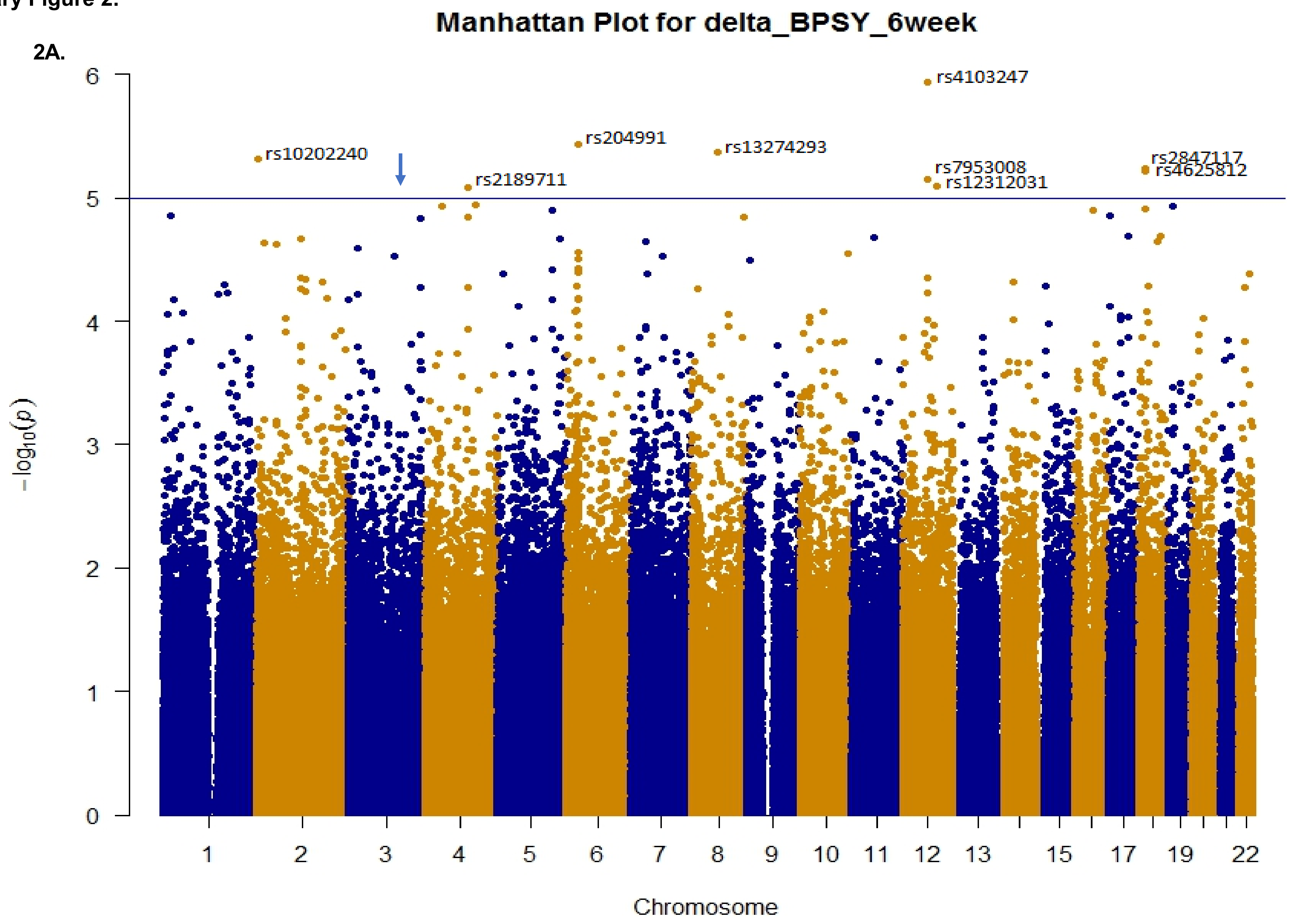

Supplementary Figure 2.

2B.

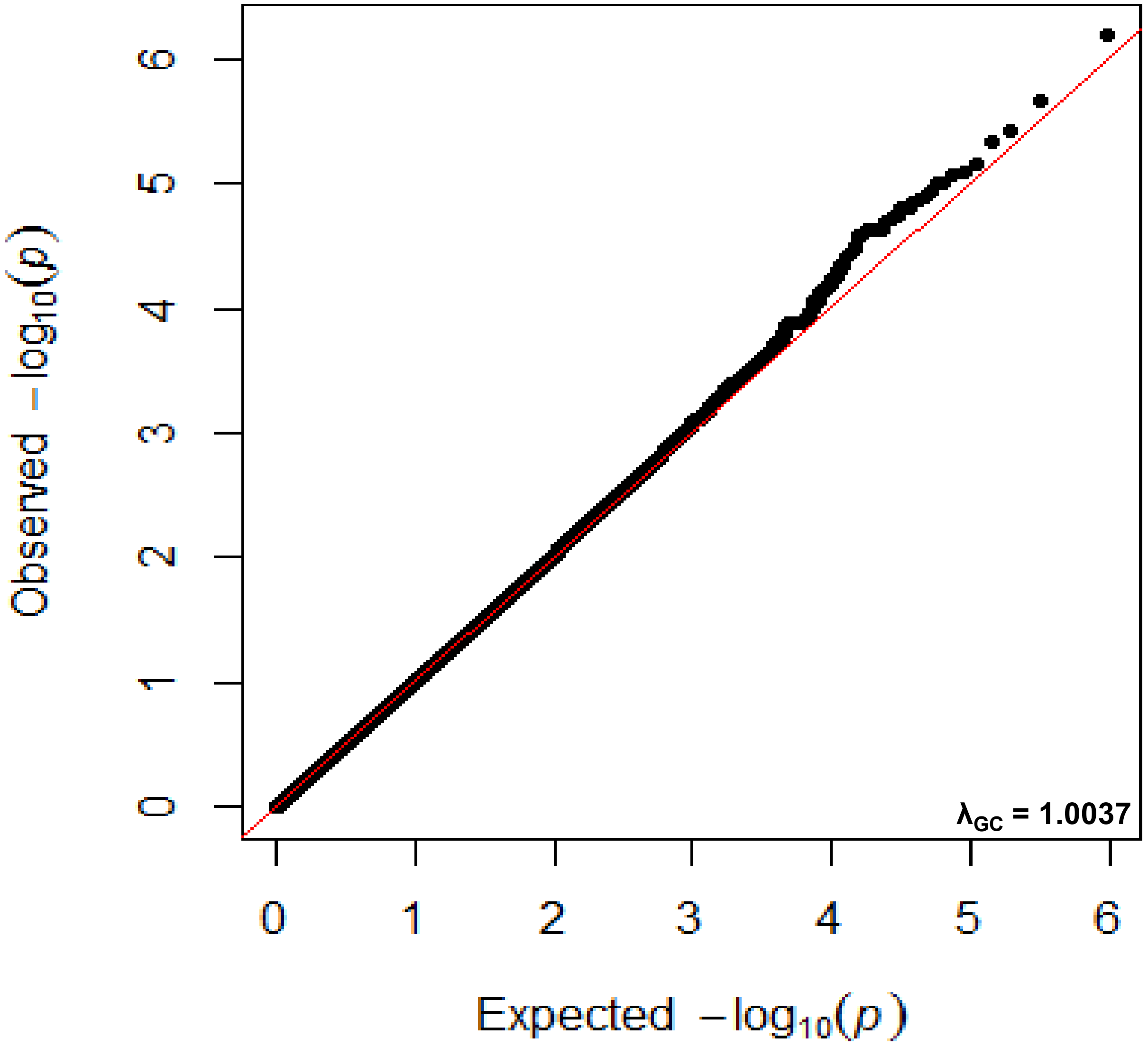

Supplementary Figure 3.

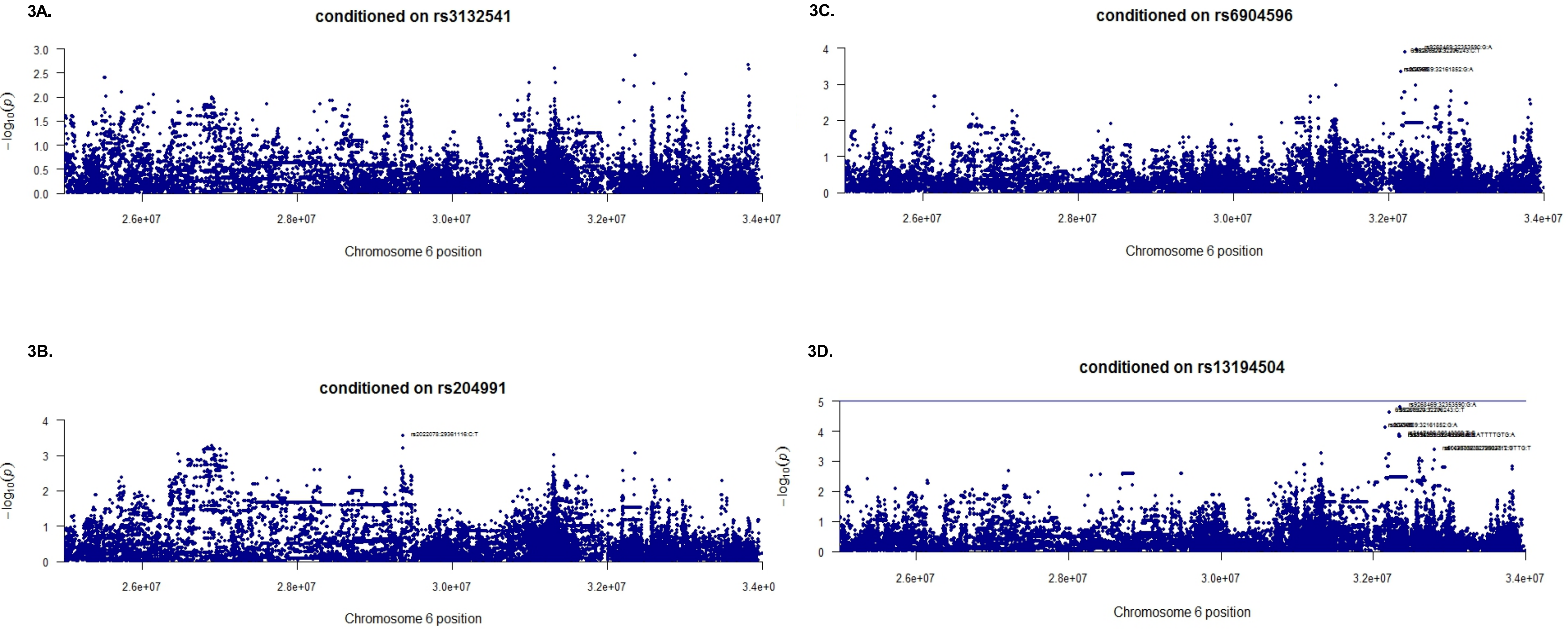

Supplementary Figure 4.

SNP1	BP1	SNP2	BP2	BETA_INT	STAT	P
rs3132541	31098734	rs204991	32161366	5.241	5.482	0.019
rs9467772	26496578	rs147925578	26937830	3.053	4.822	0.028
rs886423	30782205	rs204991	32161366	4.071	4.550	0.033
rs144022448	27456052	rs9257566	29144532	3.077	4.051	0.044
rs6904596	27491299	rs9257566	29144532	3.077	4.051	0.044
rs9467772	26496578	rs144022448	27456052	2.845	4.027	0.045
rs9467772	26496578	rs6904596	27491299	2.845	4.027	0.045
rs9467772	26496578	rs59134830	27607111	2.283	3.864	0.049

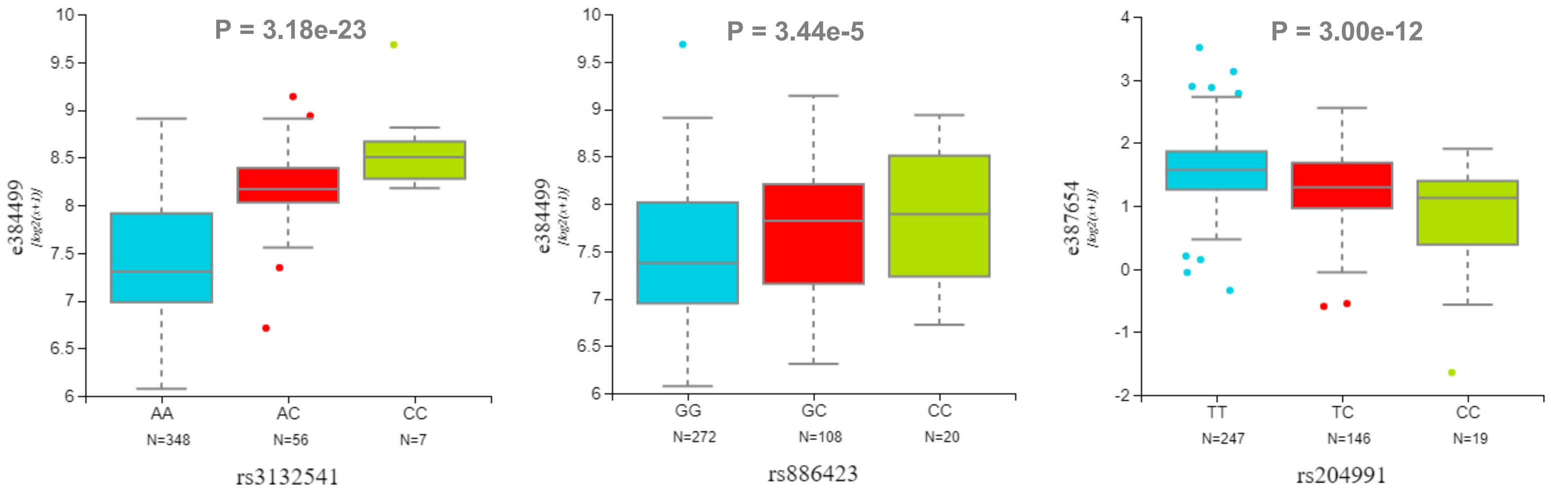

HLA-C in DLPFC **HLA-C in DLPFC** **C4A in DLPFC**

Supplementary Figure 5.

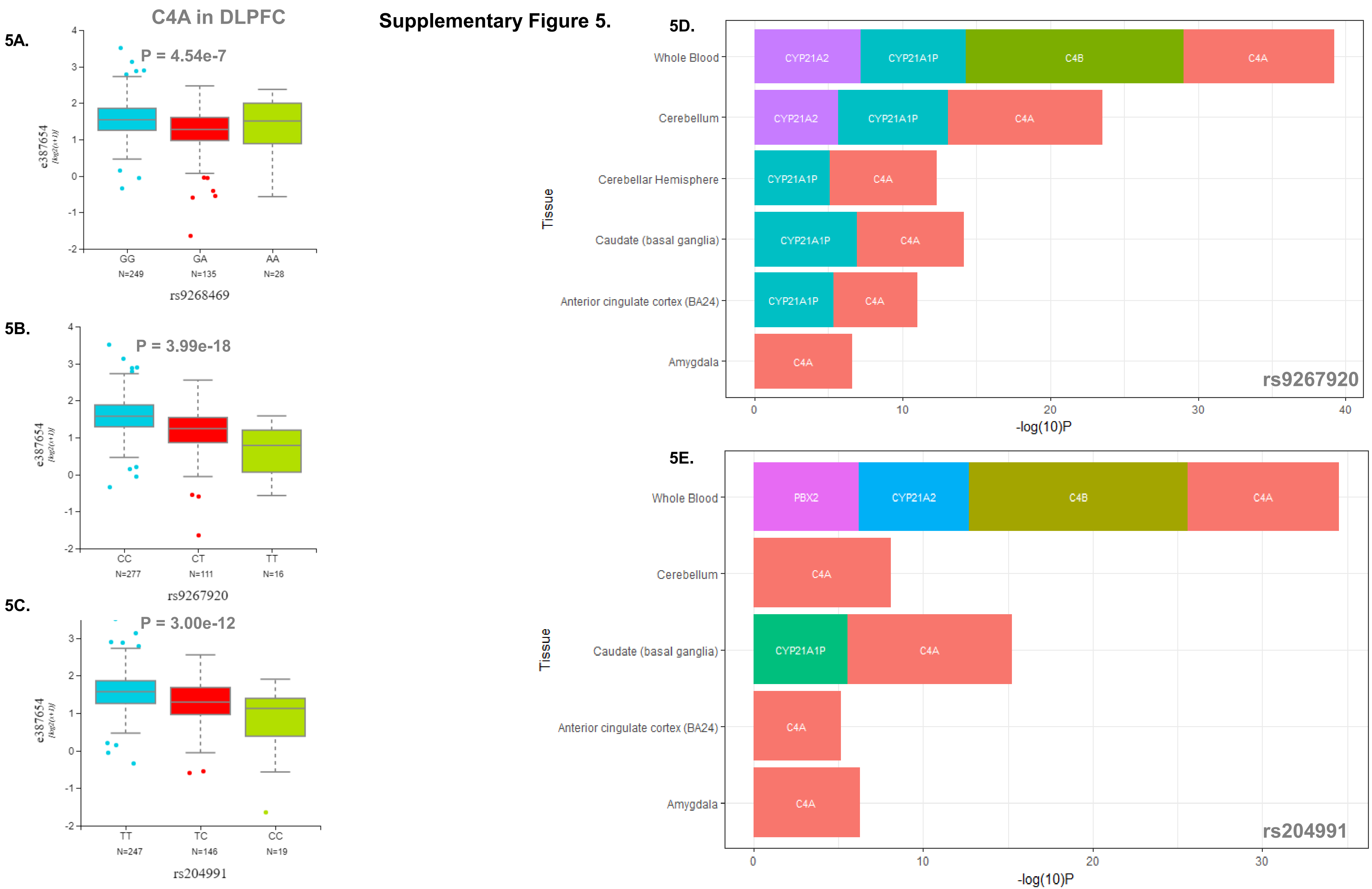

Supplementary Table 1. A summary of the sample and effect sizes of the top signal, rs204991, associated with improvement in psychotic symptoms at 6-week with different BPSY baseline cutoffs in the discovery cohort. Different cutoff values for BPSY had been implemented to avoid selection bias, and BPSY ≥ 6 gave the strongest signal compared to BPSY ≥0, ≥2, ≥4, and ≥8, with 88 out of previously reported 174 patients included in the discovery cohort. This power analysis for the linear regression with different BPSY baseline cutoff was conducted without adjustment for the covariates. 21 of 174 cases did not have data at 6WK but have data at 6MON or longer.

Supplementary Table 2. Comparing the summary statistics of association between classical HLA alleles and symptom improvement in BPSY with the summary statistics of association between those HLA alleles and the risk for schizophrenia (PGC GWAS 2009). We imputed classical HLA class I and II alleles, which include HLA-A, HLA-C, HLA-B, HLA-DRB1, HLA-DQA1, HLA-DQB1, HLA-DPA1, and HLA-DPB1 by SNP2HLA. HLA alleles were excluded from the analysis if the r^2 imputation quality score (INFO) below 0.4. When testing for association with imputed classical HLA alleles, we defined a series of binary markers coding the presence (P) or absence (A) of the allele being tested, and each different allele was tested as a biallelic position just like a SNP. The output dosage files for the HLA alleles were used for the following association testing. The linear regression assuming an additive model for minor allele, adjusted for the covariates including gender, drug, and PC 1-3 derived from the initial GWAS were conducted by PLINK to determine the association between HLA alleles or AA changes and treatment response to APDs. The original p value was provided without correction for the multiple testing as the purpose of this study is to determine if the candidate HLA alleles, previously reported to show the increased risk for SCZ, also contribute to the variation of treatment response/resistance.

Supplementary Table 3. Identified genetic risk for treatment response at xMHC also were linked to other schizophrenia related intermediate phenotypes such as cognition and brain structure. The candidate SNPs for cognition and brain structure were reported by Walters, et al. Am J Psychiatry 2013. * represents the SNP which is imputed but not genotyped in replication cohort.

Supplementary Table 4. The traits from UK BioBank were associated with the lead variants in this study using a PheWAS approach. We only listed the top three traits based on the association p value for each variant.

Supplementary Table 5. List of phenotypes from GWAS catalogue linked to the lead variants associated with treatment response

Supplementary Table 1.

BPSY Baseline Cutoff	Sample size (n)	Regression Coefficient	Variance (SE)	Significance (P value)
BPSY≥0	153	1.701	0.567	0.003
BPSY≥2	137	1.666	0.582	0.005
BPSY≥4	123	2.16	0.61	5.63E-04
BPSY≥6	88	3.832	0.786	4.87E-06
BPSY≥8	74	3.831	0.848	2.39E-05

Supplementary Table 2.

Amino Acids	Information			Discovery dataset (n = 88)					Replication dataset (n = 42)		
				∆BPSY_6WK			∆BPSY_6MON		∆BPSY_6WK (n = 42)		
	BP	A1	A2	MAF	BETA	P	BETA	P	MAF	BETA	P
AA_DRB1_74_32659926_AL	32551947	P	A	0.068	4.837	3.732E-05	1.828	0.157	0.155	1.726	0.023
AA_DRB1_74_32659926_QE	32551947	P	A	0.068	4.837	3.732E-05	1.828	0.157	0.262	1.708	0.005
AA_DRB1_77_32659917	32551938	N	T	0.068	4.837	3.732E-05	1.828	0.157	0.155	1.726	0.023
AA_DRB1_71_32659935_E	32551956	P	A	0.080	4.093	2.251E-04	0.959	0.424	0.274	1.235	0.055
AA_DQB1_-10_32742295_A	32634316	P	A	0.108	3.147	1.742E-03	1.912	0.073	0.202	1.325	0.074
AA_DQB1_-10_32742295_S	32634316	A	P	0.108	3.147	1.742E-03	1.912	0.073	0.202	1.325	0.074
AA_DQB1_28_32740753	32632774	S	T	0.108	3.147	1.742E-03	1.912	0.073	0.202	1.325	0.074
AA_DQB1_30_32740747_Y	32632768	P	A	0.108	3.147	1.742E-03	1.912	0.073	0.202	1.325	0.074
AA_DQB1_37_32740726_D	32632747	A	P	0.108	3.147	1.742E-03	1.912	0.073	0.214	1.319	0.075
AA_DQB1_37_32740726_Y	32632747	P	A	0.108	3.147	1.742E-03	1.912	0.073	0.202	1.325	0.074
AA_DQB1_46_32740699	32632720	E	V	0.108	3.147	1.742E-03	1.912	0.073	0.202	1.325	0.074
AA_DQB1_47_32740696	32632717	F	Y	0.108	3.147	1.742E-03	1.912	0.073	0.202	1.325	0.074
AA_DQB1_52_32740681	32632702	L	P	0.108	3.147	1.742E-03	1.912	0.073	0.202	1.325	0.074
AA_DQB1_55_32740672_L	32632693	P	A	0.108	3.147	1.742E-03	1.912	0.073	0.202	1.325	0.074
AA_DQB1_71_32740624_A	32632645	P	A	0.108	3.147	1.742E-03	1.912	0.073	0.202	1.325	0.074
AA_DQB1_74_32740615_A	32632636	P	A	0.108	3.147	1.742E-03	1.912	0.073	0.202	1.325	0.074
AA_DRB1_67_32659947_F	32551968	P	A	0.324	1.967	1.809E-03	0.180	0.798	0.464	0.512	0.390
AA_C_66_31347429	31239449	N	K	0.159	2.455	2.228E-03	1.141	0.216	0.702	0.653	0.322
AA_B_45_31432581_KG	31324601	A	P	0.347	-1.960	2.651E-03	-1.800	0.010	0.321	-0.602	0.342
AA_B_45_31432581_M	31324601	P	A	0.347	-1.960	2.651E-03	-1.800	0.010	0.321	-0.602	0.342
AA_B_194_31431315_I	31323335	P	A	0.409	-1.640	5.128E-03	-1.528	0.017	0.369	-0.629	0.293
AA_B_194_31431315_V	31323335	A	P	0.409	-1.640	5.128E-03	-1.528	0.017	0.369	-0.629	0.293
AA_DRB1_32_32660052	32552073	H	Y	0.171	2.281	5.265E-03	1.029	0.234	0.357	1.038	0.108
AA_B_67_31432515_S	31324535	A	P	0.421	-1.622	6.279E-03	-1.342	0.042	0.464	-0.350	0.555
AA_B_45_31432581_KM	31324601	A	P	0.398	-1.585	7.219E-03	-1.359	0.037	0.357	-0.615	0.298
AA_B_45_31432581_TG	31324601	A	P	0.398	-1.585	7.219E-03	-1.359	0.037	0.357	-0.615	0.298
**rs3129996**	**30651586**	**A**	**C**	**0.074**	**-0.999**	**0.405**	**-0.537**	**0.685**	**0.095**	**0.449**	**0.685**
**AA_A_62_30018696_GE**	**29910716**	**P**	**A**	**0.432**	**0.914**	**0.177**	**-1.118**	**0.113**	**0.524**	**0.605**	**0.291**
**AA_A_66_30018708**	**29910728**	**N**	**K**	**0.438**	**0.715**	**0.283**	**-0.981**	**0.156**	**0.726**	**0.565**	**0.415**

Supplementary Table 3.

SNP_ID	BP_hg19	Alleles		Discovery dataset (n = 88)			Replication dataset (n = 42)		
		Minor	Major	BETA	SE	P_value	BETA	SE	P_value
rs6904071*	27047256	A	G	1.938	0.798	0.017	1.361	0.926	0.150
rs13219354*	27185664	C	T	2.424	0.856	0.006	1.697	1.021	0.105
rs3131296	32172993	T	C	4.726	1.072	3.11E-05	1.504	0.960	0.126
rs6932590*	27248931	C	T	1.225	0.633	0.056	0.499	0.857	0.564

Supplementary Table 4.

SNP ID	Study ID	Trait	P-value	Beta	Odds Ratio	N Cases/ N Overall
rs204991	NEALEUKB_20002_1225	Non-cancer illness code, self-reported: hyperthyroidism/thyrotoxicosis	1.64E-42	0.463	1.589	2547/337159
rs3132541			2.13E-71	0.693	1.999	
rs6904596			1.18E-23	0.421	1.524	
rs204991	NEALEUKB_20002_1456	Non-cancer illness code, self-reported: malabsorption/coeliac disease	5.56E-309	1.995	7.350	1452/337159
rs3132541			5.56E-309	2.105	8.204	
rs6904596			4.61E-148	1.440	4.222	
rs204991	NEALEUKB_K90	Diagnoses - main ICD10: K90 Intestinal malabsorption	1.60E-138	1.712	5.542	624/337159
rs3132541			3.39E-120	1.823	6.188	
rs6904596			5.14E-39	1.107	3.025	

Supplementary Table 5.

Lead Variant rsID	Study ID	Trait	Lead Variant P-value	PMID	Author (Year)	Study N	LD (r^2)	Is in 95% Credible Set
rs204990	GCST005008	Scarlet fever	7.00E-09	28928442	Tian C	120649	1	
rs3131010	GCST004521	Autism spectrum disorder or schizophrenia	7.00E-14	28540026	Anney RJL	15954	0.891937	
rs1793894	GCST004521	Autism spectrum disorder or schizophrenia	3.00E-17	28540026	Anney RJL	15954	0.792648	
rs3130908	GCST004521	Autism spectrum disorder or schizophrenia	5.00E-15	28540026	Anney RJL	15954	0.764338	
rs3094005	GCST004365	Blood protein levels	4.00E-38	28240269	Suhre K	1335	0.764338	
rs2442735	GCST006101	Cardiometabolic and hematological traits	2.00E-46	27668658	Iotchkova V	138486	0.802992	
rs3131781	GCST005902	Depression (broad)	9.00E-09	29662059	Howard DM	322580	0.848482	
rs2523607	GCST002636	Diffuse large B cell lymphoma	2.00E-10	25261932	Cerhan JR	17439	0.72729	
rs3130544	GCST001779	Hematology traits	5.00E-07	23263863	Li J	14177	0.738826	
rs7750641	GCST000984	Idiopathic membranous nephropathy	3.00E-58	21323541	Stanescu HC	2894	0.974293	
rs3134792	GCST000984	Idiopathic membranous nephropathy	6.00E-72	21323541	Stanescu HC	2894	0.813905	
rs3130544	GCST000984	Idiopathic membranous nephropathy	9.00E-58	21323541	Stanescu HC	2894	0.948736	
rs1639108	GCST004748	Lung cancer	8.00E-17	28604730	McKay JD	85716	0.94951	
rs1634721	GCST004748	Lung cancer	3.00E-17	28604730	McKay JD	85716	0.848482	
rs886422	GCST004748	Lung cancer	7.00E-19	28604730	McKay JD	85716	0.753351	
rs3094222	GCST004748	Lung cancer	9.00E-17	28604730	McKay JD	85716	0.987201	
rs2596500	GCST004749	Lung cancer in ever smokers	2.00E-15	28604730	McKay JD	40187	0.836473	
rs886422	GCST004749	Lung cancer in ever smokers	2.00E-13	28604730	McKay JD	40187	0.753351	
rs2922994	GCST002742	Marginal zone lymphoma	2.00E-09	25569183	Vijai J	8408	0.802992	
rs3130544	GCST001611	Myasthenia gravis	2.00E-90	23055271	Gregersen PK	9569	0.948736	
rs2734583	GCST003092	Myositis	3.00E-49	26291516	Miller FW	6434	0.741285	
rs3131618	GCST003092	Myositis	2.00E-45	26291516	Miller FW	6434	0.835614	
rs3131619	GCST003092	Myositis	3.00E-45	26291516	Miller FW	6434	0.812563	
rs3130614	GCST003092	Myositis	4.00E-48	26291516	Miller FW	6434	0.786769	
rs9267488	GCST003092	Myositis	6.00E-49	26291516	Miller FW	6434	0.741285	
rs3094013	GCST003092	Myositis	2.00E-45	26291516	Miller FW	6434	0.812563	
rs1800628	GCST003858	Oral cavity cancer	7.00E-08	27749845	Lesseur C	9575	0.750959	
rs3132541	GCST003393	Parental longevity (mother's age at death)	0.000005	27015805	Pilling LC	52776	1	
rs1634726	GCST003393	Parental longevity (mother's age at death)	0.000006	27015805	Pilling LC	52776	0.848482	
rs2263298	GCST005009	Pneumonia	3.00E-09	28928442	Tian C	130639	0.764435	
rs1619179	GCST005009	Pneumonia	3.00E-10	28928442	Tian C	130639	0.961769	
rs3130631	GCST005009	Pneumonia	2.00E-09	28928442	Tian C	130639	0.729126	
rs3094013	GCST006052	Polymyositis	6.00E-76	26362759	Rothwell S	16582	0.812563	
rs3134792	GCST000165	Psoriasis	1.00E-09	18364390	Capon F	4894	0.813905	
rs2596565	GCST002357	Rheumatoid arthritis (ACPA-negative)	9.00E-09	24532677	Bossini-Castillo L	9009	0.848142	
rs3130614	GCST005790	Rosacea symptom severity	3.00E-10	29771307	Aponte JL	73265	0.786769	
rs4143333	GCST005790	Rosacea symptom severity	1.00E-09	29771307	Aponte JL	73265	0.790527	
rs886420	GCST005541	Sarcoidosis (Lofgren's syndrome vs non-Lofgren's syndrome)	2.00E-24	26651848	Rivera NV	1525	0.764435	
rs2596500	GCST003048	Schizophrenia	8.00E-20	26198764	Goes FS	151161	0.836473	
rs3094087	GCST004746	Small cell lung carcinoma	1.00E-07	28604730	McKay JD	24108	0.791344	
rs1619179	GCST004750	Squamous cell lung carcinoma	4.00E-15	28604730	McKay JD	63053	0.961769	
rs1634726	GCST004750	Squamous cell lung carcinoma	6.00E-16	28604730	McKay JD	63053	0.848482	
rs1264304	GCST004750	Squamous cell lung carcinoma	3.00E-16	28604730	McKay JD	63053	0.753351	
rs3132510	GCST005006	Tuberculosis	4.00E-14	28928442	Tian C	88716	0.872558	
rs114046333	GCST005006	Tuberculosis	1.00E-14	28928442	Tian C	88716	0.761412	
rs3095156	GCST005012	Urinary tract infection frequency	0.000007	28928442	Tian C	68478	0.775782	
rs3094013	GCST004567	Waist-to-hip ratio adjusted for BMI (joint analysis for main effect and physical activity interaction)	8.00E-07	28448500	Graff M	199748	0.764769	
rs3094013	GCST004578	Waist-to-hip ratio adjusted for BMI in active individuals	0.000002	28448500	Graff M	151260	0.776316	
rs3094013	GCST004576	Waist-to-hip ratio adjusted for body mass index	5.00E-08	28448500	Graff M	199748	0.764769	
rs141342723	GCST004521	Autism spectrum disorder or schizophrenia	4.00E-24	28540026	Anney RJL	15954	0.866971	
rs13212562	GCST004521	Autism spectrum disorder or schizophrenia	3.00E-16	28540026	Anney RJL	15954	0.734101	
rs45509595	GCST004988	Breast cancer	5.00E-09	29059683	Michailidou K	243019	0.801324	
rs55834529	GCST004988	Breast cancer	3.00E-09	29059683	Michailidou K	243019	0.750074	
rs34196306	GCST004988	Breast cancer	1.00E-09	29059683	Michailidou K	243019	0.739722	
rs56114371	GCST004988	Breast cancer	2.00E-09	29059683	Michailidou K	243019	0.762442	
rs55834529	GCST005902	Depression (broad)	3.00E-09	29662059	Howard DM	322580	0.783105	
rs35715914	GCST005902	Depression (broad)	8.00E-11	29662059	Howard DM	322580	0.866971	
rs483143	GCST005902	Depression (broad)	8.00E-12	29662059	Howard DM	322580	0.875697	
rs7749305	GCST005902	Depression (broad)	4.00E-11	29662059	Howard DM	322580	0.987627	

Lead Variant rsID	Study ID	Trait	Lead Variant P-value	PMID	Author (Year)	Study N	LD (r^2)	Is in 95% Credible Set
rs35741362	NEALEUKB_3063	Forced expiratory volume in 1-second (FEV1)	1.78E-18		Neale et al.	307638		0.049727174
rs6904596	NEALEUKB_20150	Forced expiratory volume in 1-second (FEV1), Best measure	5.17E-15		Neale et al.	255492	1	0.049677764
rs6904596	NEALEUKB_20154	Forced expiratory volume in 1-second (FEV1), predicted percentage	3.31E-20		Neale et al.	110423	1	0.084774243
rs483143	NEALEUKB_2257	Hearing difficulty/problems with background noise	1.35E-09		Neale et al.	330759	0.875697	
rs13212651	GCST005316	Intelligence (MTAG)	6.00E-13	29326435	Hill WD	248482	0.854637	
rs13217620	GCST004748	Lung cancer	1.00E-10	28604730	McKay JD	85716	0.750474	
rs35037868	GCST004748	Lung cancer	2.00E-10	28604730	McKay JD	85716	0.854637	
rs149866169	GCST004748	Lung cancer	2.00E-11	28604730	McKay JD	85716	0.987627	
rs35768595	GCST004748	Lung cancer	5.00E-09	28604730	McKay JD	85716	0.794715	
rs13199649	GCST004748	Lung cancer	2.00E-10	28604730	McKay JD	85716	0.807572	
rs13212562	GCST004749	Lung cancer in ever smokers	1.00E-08	28604730	McKay JD	40187	0.734101	
rs13217620	GCST004749	Lung cancer in ever smokers	3.00E-08	28604730	McKay JD	40187	0.750474	
rs13218875	GCST004749	Lung cancer in ever smokers	1.00E-08	28604730	McKay JD	40187	0.807572	
rs149866169	GCST004749	Lung cancer in ever smokers	8.00E-09	28604730	McKay JD	40187	0.987627	
rs35768595	GCST004749	Lung cancer in ever smokers	1.00E-07	28604730	McKay JD	40187	0.794715	
rs34864796	GCST004183	Lung function (FEV1)	6.00E-14	28166213	Wain LV	144318	0.987627	
rs34864796	GCST004185	Lung function (FEV1/FVC)	4.00E-10	28166213	Wain LV	144318	0.987627	
rs34864796	GCST004184	Lung function (FVC)	5.00E-10	28166213	Wain LV	144318	0.987627	
rs149866169	GCST005326	Neuroticism (MTAG)	4.00E-10	29292387	Turley P	168105	0.987627	
rs200949	NEALEUKB_20002_1226	Non-cancer illness code, self-reported: hypothyroidism/myxoedema	2.95E-12		Neale et al.	337159	0.83358	0.005028503
rs34788973	NEALEUKB_884	Number of days/week of moderate physical activity 10+ minutes	7.78E-09		Neale et al.	321309	0.807572	0.010837588
rs13195040	GCST003871	QRS complex (Cornell)	3.00E-08	27659466	van der Harst P	80347	0.787097	
rs10484399	GCST005541	Sarcoidosis (Lofgren's syndrome vs non-Lofgren's syndrome)	1.00E-16	26651848	Rivera NV	1525	0.855205	
rs34706883	GCST003048	Schizophrenia	5.00E-27	26198764	Goes FS	151161	0.854637	
rs55834529	GCST003048	Schizophrenia	9.00E-27	26198764	Goes FS	151161	0.783105	
rs17693963	GCST001565	Schizophrenia	3.00E-11	22688191	Bergen SE	30518	0.703327	
rs17693963	GCST002295	Schizophrenia or bipolar disorder	3.00E-11	24280982	Ruderfer DM	39202	0.703327	
rs17693963	GCST002254	Schizophrenia, schizoaffective disorder or bipolar disorder	2.00E-09	24166486	Sleiman P	50722	0.703327	
rs200949	NEALEUKB_2100	Seen a psychiatrist for nerves, anxiety, tension or depression	3.64E-10		Neale et al.	335888	0.83358	
rs13218875	GCST004750	Squamous cell lung carcinoma	2.00E-10	28604730	McKay JD	63053	0.807572	
rs71537559	GCST004750	Squamous cell lung carcinoma	3.00E-11	28604730	McKay JD	63053	0.855205	
rs149866169	GCST004750	Squamous cell lung carcinoma	1.00E-11	28604730	McKay JD	63053	0.987627	
rs35768595	GCST004750	Squamous cell lung carcinoma	9.00E-10	28604730	McKay JD	63053	0.794715	
rs149866169	GCST005325	Subjective well-being (MTAG)	3.00E-10	29292387	Turley P	388538	0.987627	
rs200949	LEUKB_20003_114119	Treatment/medication code: levothyroxine sodium	1.71E-14		Neale et al.	337159	0.83358	0.006062046
rs13191227	GCST005012	Urinary tract infection frequency	3.00E-07	28928442	Tian C	68478	0.842865	
rs35491132	GCST005012	Urinary tract infection frequency	0.000002	28928442	Tian C	68478	0.866971	
rs55834529	GCST005012	Urinary tract infection frequency	0.000001	28928442	Tian C	68478	0.783105	
rs9468350	NEALEUKB_20160	Ever smoked	3.83E-08		Neale et al.	336067		0.000837601
rs17312661	NEALEUKB_78	Heel bone mineral density (BMD) T-score, automated	3.14E-10		Neale et al.	194398		0.003527329
rs34661125	GCST004748	Lung cancer	2.00E-12	28604730	McKay JD	85716	0.707621	
rs67340775	GCST004749	Lung cancer in ever smokers	7.00E-10	28604730	McKay JD	40187	0.719691	
rs9380069	NEALEUKB_6177_3	Medication for cholesterol, blood pressure or diabetes: Insulin	1.07E-09		Neale et al.	154702		0.000778512
rs17301128	NEALEUKB_6149_1	Mouth/teeth dental problems: Mouth ulcers	1.95E-08		Neale et al.	336138		0.00304548
rs13217619	GCST003048	Schizophrenia	2.00E-29	26198764	Goes FS	151161	0.709137	
rs67340775	NEALEUKB_2090	Seen doctor (GP) for nerves, anxiety, tension or depression	2.56E-12		Neale et al.	335108	0.719691	
rs34878803	GCST004750	Squamous cell lung carcinoma	4.00E-11	28604730	McKay JD	63053	0.707621	
rs67998226	LEUKB_20003_114088	Treatment/medication code: insulin product	3.36E-16		Neale et al.	337159	0.707621	0.003022138
rs9267920	GCST004521	Autism spectrum disorder or schizophrenia	3.00E-15	28540026	Anney RJL	15954	1	
rs3134942	GCST004365	Blood protein levels	1.00E-16	28240269	Suhre K	1335	0.716798	
rs3131296	GCST000435	Schizophrenia	2.00E-10	19571808	Stefansson H	47536	0.716798	
rs3117103	GCST004521	Autism spectrum disorder or schizophrenia	1.00E-14	28540026	Anney RJL	15954	0.796363	
rs9268510	GCST004521	Autism spectrum disorder or schizophrenia	3.00E-11	28540026	Anney RJL	15954	0.723461	
rs3129843	GCST006053	Dermatomyositis or juvenile dermatomyositis	2.00E-48	26362759	Rothwell S	16530	0.712194	
rs3129843	GCST006051	Idiopathic inflammatory myopathy	9.00E-133	26362759	Rothwell S	18207	0.712194	
rs3129966	GCST004748	Lung cancer	2.00E-17	28604730	McKay JD	85716	0.723461	
rs2395160	GCST004749	Lung cancer in ever smokers	8.00E-11	28604730	McKay JD	40187	0.723461	
rs2894254	GCST005790	Rosacea symptom severity	1.00E-13	29771307	Aponte JL	73265	0.723461	
rs3135394	GCST005541	Sarcoidosis (Lofgren's syndrome vs non-Lofgren's syndrome)	3.00E-33	26651848	Rivera NV	1525	0.712194	
rs3135394	GCST004878	Sjögren's syndrome	5.00E-113	24097067	Lessard CJ	8392	0.712194	
rs3117109	GCST004750	Squamous cell lung carcinoma	6.00E-15	28604730	McKay JD	63053	0.723461	
rs3135394	GCST004867	Systemic lupus erythematosus	2.00E-60	19838195	Gateva V	15461	0.712194	
